# The explanation of educational disparities in adiposity by lifestyle, socioeconomic and mental health mediators: a multiple mediation model

**DOI:** 10.1038/s41430-024-01403-1

**Published:** 2024-01-20

**Authors:** Anna Bartoskova Polcrova, Albert J. Ksinan, Juan P. González-Rivas, Martin Bobak, Hynek Pikhart

**Affiliations:** 1 RECETOX, Faculty of Science, Masaryk University, Kotlarska 2, Brno, Czech Republic; 2 International Clinical Research Centre (ICRC), St Anne’s University Hospital Brno (FNUSA), Brno, Czech Republic; 3 Department of Global Health and Population. Harvard TH Chan School of Public Health, Harvard University, Boston, MA USA; 4 Research Department of Epidemiology and Public Health, University College London, London, UK

**Keywords:** Risk factors, Epidemiology

## Abstract

**Background:**

The inverse association between education and obesity was previously found in numerous studies. This study aims to assess several possible mediators in the educational disparities in adiposity. We hypothesize the potential mediating role of lifestyle, socioeconomic, and mental health factors in the association between education and adiposity.

**Methods:**

Cross-sectional population-based sample from Czechia included 2,154 25-64 years old subjects (54.6% women). Education was classified as high, middle, and low. Adiposity was assessed as a latent variable based on body fat percentage, BMI, waist circumference, and visceral fat. The mediation potential of unhealthy dietary behavior, alcohol intake, smoking, sedentary behaviors, income, stress, depression, and quality of life was assessed in age-adjusted sex-specific multiple mediation models.

**Results:**

The negative direct effect of education on adiposity was statistically significant at 5% level of significance in both sexes. For men, the indirect effect was statistically significant via sedentary behavior (β = 0.041; 95% CI [0.025–0.062]) with a mediation ratio of 23.7%. In women, the indirect effect was statistically significant via dietary risk (β = −0.023, 95% CI [−0.037, −0.013]), alcohol intake (β = −0.006; 95% CI [−0.014, −0.001]), sedentary behavior (β = 0.012, 95% CI [0.004,0.023]), income (β = −0.022; 95% CI [−0.041, −0.004]), and mental health (β = −0.007; 95% CI [−0.019, −0.001]). The total mediation ratio in women was 30.5%.

**Conclusions:**

Sedentary behaviors had mediating role in the association between education and adiposity in both sexes, with more important role in men. In addition, unhealthy diet and lower income partially mediated the educational gradient in adiposity in women.

## Introduction

Cardiovascular diseases persist as the leading cause of death in Czechia despite the reduction of cardiovascular mortality in the last 30 years [1]. On the contrary, the prevalence of cardiometabolic drivers such as obesity, prediabetes, and diabetes has neither increased nor decreased in the past [1]. Poor cardiometabolic health is associated with a lower level of education [2–4], which is also considered to be the strongest determinant of cardiovascular mortality in the Czech population [5]. Previous study [2] including 7081 Czech participants aged 45–69 years, described the strong association between a low level of education and obesity in both sexes. The negative association between socioeconomic position and prevalence of obesity was found in several high-income countries [6]. Education level per se is not likely to be directly related to the risk of obesity. For this reason, it is necessary to focus on factors on potential causal pathway linking educational disadvantage with increased risk of obesity. Lower educational levels have been shown to be associated with poor lifestyle [7, 8], socioeconomic disadvantage [9], and mental health problems [10], which were in turn all shown to be risk factors for increased adiposity [8, 11–13]. Moreover, there are previously described sex differences that must be considered. Women have a physiologically higher percentage of body fat than men and tend to have lower education and income. On the contrary, men are more likely to be obese than women [14]. Women and men also differ in their diets, cravings, and adherence to dietary recommendations [15]. Based on previous evidence, we aim to investigate the educational disparities in adiposity and the role of potential mediators in these educational disparities in large community sample of middle-aged men and women from Brno region in the Czech Republic. Furthermore, we want to investigate whether the role of identified mediators may differ between men and women. We hypothesize the potential mediating role of lifestyle factors, socioeconomic characteristics, and mental health factors in the association between education and increased adiposity. We further hypothesize that women may have wider variety of mediators influencing the association between education and adiposity when compared to men.

## Methods

### Design and population

Data from the Kardiovize study [16] were used. The Kardiovize study is an epidemiological study including a random sample of adult residents of the city of Brno, the second-largest city in Czechia, with 373,327 residents. Survey sampling was done in January 2013 with technical assistance from the health insurance companies. A random age and sex-stratified sample of 2154 men and women has been enrolled in the study. No information on non-respondents was available due to confidentiality restrictions.

### Data collection

In-person health interviews were performed by trained nurses and physicians at the International Clinical Research Center of the St Anne’s University Hospital in Brno. The questionnaire included demographics, socioeconomic characteristics, cardiovascular risk behaviors, smoking status, medical history, and mental health [16]. The anthropometric assessment included height and weight measurements using a medical digital scale with a meterstick (SECA 799®; SECA, GmbH and Co. KG, Germany) and manual tape measurement of the waist circumference. Weight and body composition analyses were performed using a scale with bioelectrical impedance analysis capabilities (InBody 370; BIOSPACE Co., Ltd., Korea).

### Measures

#### Predictor and outcome

##### Education

Educational attainment was classified into three groups: “high”, including subjects with higher professional or university education; “middle”, defined as high school education; and “low”, defined as elementary or vocational education without a final graduation exam.

##### Adiposity

The latent variable adiposity was constructed based on four main available adiposity biomarkers - body fat percentage, body mass index, waist circumference, and visceral fat. All adiposity biomarkers were assessed as continuous variables. The adequacy of the unidimensional latent factor was confirmed by CFA analysis.

#### Potential mediators

##### Dietary risk

Dietary risk patterns were assessed using a dietary risk score derived from the 43-item Food Frequency Questionnaire. Participants were asked to indicate the frequency of consumption of specific food groups in the past week on a scale including 10 options from “almost never” to “six or more times a day”. In total, six specific risky dietary patterns were identified based on the Global Burden of Disease [17] methodology (Table 1), and their presence was assessed. Then, risky dietary patterns were summed, and the total dietary risk score ranged from 0 to 6 points.

**Table 1:** Definition of dietary risky score items.

Diet low in fruit	Mean daily consumption of fruits (fresh, frozen, cooked, canned, or dried fruits, excluding fruit juices and salted or pickled fruits)	Less than 250 g per day
Diet low in vegetables	Mean daily consumption of vegetables (fresh, frozen, cooked, canned, or dried vegetables, excluding legumes and salted or pickled vegetables, juices, nuts, seeds, and starchy vegetables such as potatoes or corn)	Less than 360 g per day
Diet high in red meat	Mean daily consumption of red meat (beef, pork, lamb, and goat, but excluding poultry, fish, eggs, and all processed meats)	More than 23 g per day
Diet high in processed meat	Mean daily consumption of meat preserved by smoking, curing, salting, or addition ofchemical preservatives.	More than 2 g per day
Diet low in nuts and seeds	Mean daily consumption of nut and seed foods.	Less than 21 g per day
Diet low in legumes	Mean daily consumption of legumes (fresh, frozen, cooked, canned, or dried legumes)	Less than 60 g per day

##### Alcohol intake

Alcohol intake was evaluated as the total amount of ethanol in grams consumed during the week before data collection. The data were obtained from a 7-day alcohol consumption recall, where participant reported alcohol beverages consumed in 7 days before the visit [18].

##### Smoking

Smoking status was assessed by series of questions in the questionnaire, and categorized as current smokers, ex-smokers, and non-smokers. We defined current smoker as smoking either daily or less than daily, ex-smoker as having stopped smoking at least a year prior the interview, and non-smoker as having smoked fewer than 100 cigarettes in a lifetime.

##### Sedentary behaviors

Sedentary behaviors were assessed using an item asking participants to report their total sedentary time in minutes per week, obtained from the long version of the International Questionnaire of Physical Activity [19].

##### Income

The equivalized household income was calculated as a ratio of total household income and equivalent size. Household income was collected using categories defined by income ranges, and appropriate mid-value was then used in this calculation. The equivalent size is calculated by attributing a weight to all members of household in following way:1.0 for the first person and 0.5 for each subsequent person in the household. The equivalent size is the sum of the weights of all the members of a given household [20].

##### Stress

The stress was assessed using The Cohen Perceived Stress Questionnaire [21] (PSS). Participants rated 10 questions about the previous 4 weeks (e.g., “How often have you felt that you are unable to control important things in your life?”) on a five-point Likert-type scale ranging from never (0) to very often [4]. Thus, the overall PSS score ranged between 0 (low stress) and 40 (high stress).

##### Depression

Depressive symptoms were measured by the Patient Health Questionnaire [22] (PHQ-9). Participants rated 9 items (e.g., “I feel down, depressed, hopeless.”) on a four-point Likert-type scale ranging from never (0) to most of the time [3]. PHQ-9 thus ranged between 0 (no depressive symptoms) to 27 (high score of depressive symptoms).

##### Quality of life

Self-assessed quality of life was evaluated based on the question “In general, how would you rate your quality of life as a whole?” Participants answered on a scale ranging from 0 (very bad) to 100 (excellent).

### Data analysis

Data analyses were performed using STATA [23] software (version 16.0, StataCorp, College Station, TX, USA) and MPlus 8.6 [24]. All analyses were performed separately for both sexes to capture sex-specific relationships between variables. Continuous variables were described using means, and categorical variables were described using frequencies. Differences in levels of adiposity biomarkers and potential mediators by education levels were assessed by One-way ANOVA or Chi-Square test. Bivariate correlations between mediators were estimated to evaluate potential collinearity (Supplementary Table 1). As the mental health mediators were strongly intercorrelated, the latent variable of mental health was constructed. The outcome latent variable of adiposity was constructed using continuous variables of body fat percentage, body mass index, waist circumference, and visceral fat as indices (Supplementary Fig. 1). Then, a simple mediation analysis was performed, where each potential mediator was tested separately in a model with education as a predictor and adiposity as the outcome. Total, direct, and indirect effects were calculated. Next, a multiple mediation model including all potential mediators in one set was developed. In both sexes, the indirect paths of the association between education and adiposity via potential mediators were modeled. The direct and indirect effects were computed for the overall model as well as for mediating pathways separately. When computing the estimates and significance of the indirect effects, a bootstrapping procedure with 5000 resamples was performed.

## Results

### Subjects’ characteristics and adiposity outcome selection

In total, 2,154 (54.6% women) subjects were included, with a mean age of 46.7 years in men and 47.8 years in women. In men, the most prevalent level of education was high (45.0%); in women, it was middle (42.3%). Men had a higher BMI and waist circumference than women, while women had a higher body fat percentage and visceral fat area than men (*p* < 0.001). In both sexes, the mean values of adiposity biomarkers were significantly higher at lower educational levels (Table 2).

**Table 2:** Subjects characteristics and potential mediators in categories of education by sex.

	Men					Women				
	Total	High	Middle	Low	*p*	Total	High	Middle	Low	*p*
*n* (%)	977 (45.36)	440 (45.04)	333 (34.08)	204 (20.88)		1177 (54.64)	456 (38.74)	498 (42.31)	223 (18.95)	
Age (years)	46.74	45.18	46.27	50.80	**<0.001**	47.77	44.23	49.10	51.95	**<0.001**
BMI (kg/m^2^)	26.86	25.96	27.11	28.39	**<0.001**	25.51	23.97	26.10	27.39	**<0.001**
Waist circumference (cm)	96.75	94.05	97.44	101.36	**<0.001**	84.53	80.58	85.86	89.68	**<0.001**
Body fat (%)	21.61	20.09	21.81	24.60	**<0.001**	30.75	27.96	31.54	34.66	**<0.001**
VFA (cm^2^)	86.44	78.37	87.91	101.61	**<0.001**	92.01	81.54	96.16	104.24	**<0.001**
Potential mediators										
Nutrition										
Diet low in fruit (%)	78.12	75.45	76.58	86.27	**0.006**	67.23	68.20	65.26	70.85	0.304
Diet low in vegetables (%)	95.30	95.00	95.50	95.59	0.926	90.09	87.72	90.36	95.52	**0.005**
Diet low in legumes (%)	99.49	99.32	99.70	99.51	0.762	99.32	99.78	99.80	98.65	0.064
Diet low in nuts and seeds (%)	53.78	48.18	52.85	67.16	**<0.001**	43.78	38.16	41.57	60.54	**<0.001**
Diet high in processed meat (%)	93.15	93.86	91.29	94.61	0.243	88.48	86.40	89.56	91.48	0.106
Diet high in red meat (%)	76.58	76.14	75.38	79.41	0.541	56.73	55.26	56.02	61.88	0.233
Dietary risk score	4.96	4.88	4.91	5.23	**<0.001**	4.46	4.36	4.43	4.79	**<0.001**
Smoking										
Smokers (%)	25.28	22.73	34.83	36.27	**<0.001**	21.94	20.44	22.29	24.22	<**0.001**
Ex-smokers (%)	29.68	60.91	38.14	22.06		21.94	65.49	52.61	44.84	
Non-smokers (%)	45.04	16.36	27.03	41.67		56.12	14.07	25.10	30.94	
Alcohol consumption (g)^a^	115.83	116.90	112.33	118.79	0.799	46.72	53.66	47.82	30.87	**<0.001**
Sedentary behaviors (min)^b^	3026.40	3396.81	2793.21	2622.94	**<0.001**	2772.17	2851.01	2827.02	2538.20	**0.002**
Equalized household income (CZK)	22918.56	26717.39	21565.92	17039.42	**<0.001**	19014.73	22764.13	18129.19	13369.45	**<0.001**
Stress score	11.38	11.55	11.03	11.59	0.398	12.83	12.76	12.75	13.14	0.700
Depression score	2.90	2.99	2.59	3.24	**0.041**	3.66	3.55	3.41	4.43	**0.001**
Quality of life	77.56	79.36	77.67	73.39	**<0.001**	75.85	78.58	75.34	71.28	**<0.001**

Differences using One-Way ANOVA or Chi-Square test. Estimates statistically significant at *p* < 0.05 in bold.

^a^Grams of ethanol consumed in the last 7 days.

^b^Reported in total sitting time in minutes per week.

### The association between education and potential mediators

In both sexes, a lower level of education was associated with a higher prevalence of a diet low in nuts and seeds. In men, a lower level of education was also associated with a higher prevalence of a diet low in fruit, while in women, a lower level of education was associated with a higher prevalence of a diet low in vegetables (Table 2). In both sexes, a lower level of education was associated with a higher total dietary risk score, higher tobacco use, and lower sedentary behavior (*p* < 0.001). The alcohol intake was higher in those with a high level of education in women (*p* < 0.001), while there were no differences in alcohol intake across educational levels in men (Table 2). Lower education was also associated with lower income in both sexes (*p* < 0.001). The prevalence of depressive symptoms was higher in subjects with lower education of both sexes, but there were no differences in the prevalence of stress across educational levels. In both sexes, subjects with a higher level of education also reported a higher quality of life (*p* < 0.001; Table 2).

### A simple mediation of the association between education and adiposity

The direct effect of education on adiposity was significant after adjustment for age in both sexes (*p* < 0.001). In men, there was a statistically significant indirect effect via sedentary behavior in men (β = 0.042, 95% CI [0.026–0.062]), and it mediated 24.3% of the total effect. In women, the indirect effect significantly operated via dietary risk (β = −0.026, 95% CI [−0.040 to −0.015]), alcohol consumption (β = −0.005, 95% CI −0.012 to −0.001]), sedentary behavior (β = 0.012, 95% CI [0.005–0.023]), and mental health (β = −0.011, 95% CI [−0.025 to −0.003]) (Table 3).

**Table 3:** Simple mediation analysis by sex.

	Total effect		Path a^a^		Path b^b^		Indirect effect		Mediation ratio (%)
	β	95% CI	β	95% CI	β	95% CI	β	95% CI	
**Men**	−0.173	**−0.237 to −0.112**							
Dietary risk			**−0.143**	**−0.201 to −0.084**	0.040	−0.022 to 0.110	−0.006	−0.018 to 0.003	3.5
Alcohol consumption			−0.012	−0.076 to 0.052	0.012	−0.050 to 0.078	0.000	−0.004 to 0.001	0.0
Smoking			**0.078**	**0.011 to 0.145**	0.054	−0.008 to 0.116	0.004	0.000 to 0.013	2.3
Sedentary behavior			**0.243**	**0.183 to 0.304**	**0.172**	**0.109 to 0.235**	**0.042**	**0.026** to **0.062**	24.3
Equalized income			**0.299**	**0.244 to 0.348**	0.010	−0.054 to 0.068	0.003	−0.016 to 0.020	1.7
Mental health			−0.069	−0.145 to 0.010	0.051	−0.026 to 0.134	−0.003	−0.016 to 0.001	1.7
**Women**	−0.143	**−0.196 to −0.090**							
Dietary risk			**−0.157**	**−0.213 to −0.098**	0.164	**0.113 to 0.214**	**−0.026**	**−0.040 to −0.015**	18.2
Alcohol consumption			**0.104**	**0.043 to 0.162**	**−0.051**	**−0.097 to −0.003**	**−0.005**	**−0.012 to −0.001**	3.5
Smoking			**0.080**	**0.022 to 0.141**	0.020	−0.035 to 0.073	0.002	−0.002 to 0.008	1.4
Sedentary behavior			**0.092**	**0.033 to 0.151**	**0.135**	**0.079 to 0.185**	**0.012**	**0.005 to 0.023**	8.4
Equalized income			**0.335**	**0.283 to 0.383**	**−0.060**	**−0.113 to −0.001**	−0.020	−0.038 to 0.000	14.0
Mental health			**−0.122**	**−0.192 to −0.050**	**0.091**	**0.030 to 0.153**	**−0.011**	**−0.025 to −0.003**	7.7

Mediation analysis performed with each mediator separately. Results adjusted for age. Estimates statistically significant at *p* < 0.05 in bold.

^a^Path a represents the association between education as a predictor and mediator as the outcome.

^b^Path b represents the association between mediators as predictors and adiposity as the outcome.

### Multiple mediation model

**Fig. 1:**
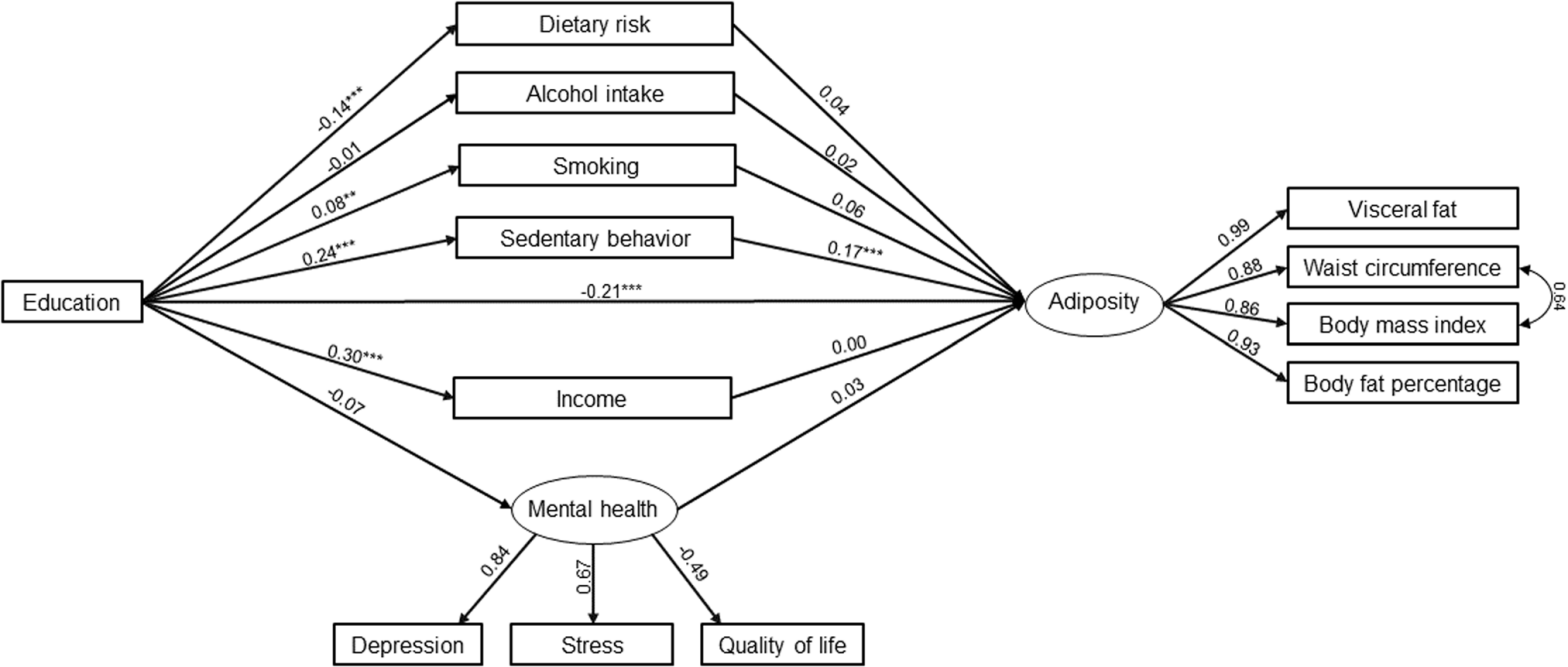
Standardized coefficients (β**)** of the multiple mediation structural equation model for men, adjusted for age. Model fit: χ^2^[62] = 319.5, *p* < 0.001, CFI = 0.959, RMSEA = 0.065, 90% CI RMSEA (0.058, 0.072). **p < 0.05. ***p < 0.001.

**Fig. 2:**
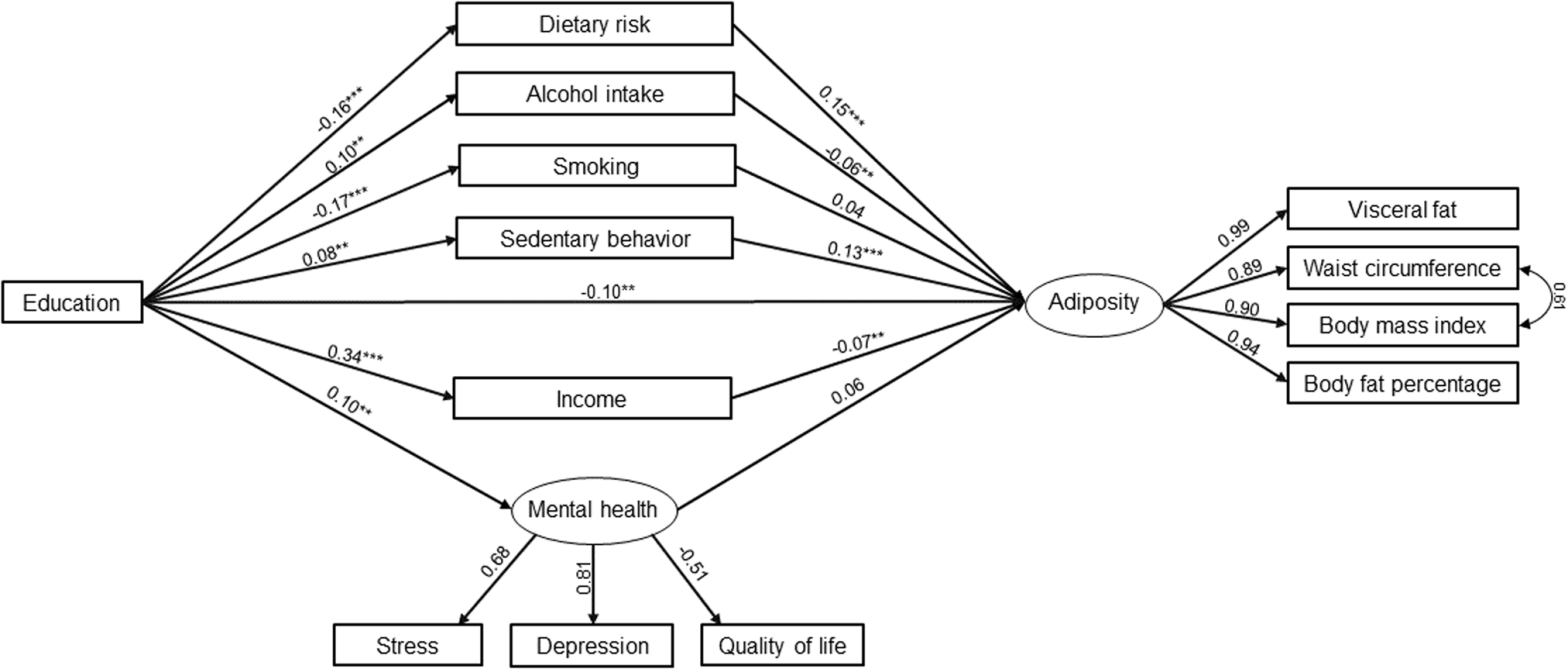
Standardized coefficients (β) of the full structural equation model for women, adjusted for age. Model fit: χ^2^[62] = 395.1, *p* < 0.001, CFI = 0.959, RMSEA = 0.067, 90% CI RMSEA (0.061, 0.074). **p < 0.05. ***p < 0.001.

Multiple mediation models are shown in Figs. 1 and 2. Observed indirect effects are summarized in Table 4. For both sexes, an adequate model fit was achieved with χ^2^[62] = 319.5, *p* < 0.001, CFI = 0.959, RMSEA = 0.065, 90% CI RMSEA [0.058, 0.072] in men and χ^2^[62] = 395.1, *p* < 0.001, CFI = 0.959, RMSEA = 0.067, 90% CI RMSEA [0.061, 0.074] in women.

**Table 4:** Standardized total, direct, and indirect effects of multiple mediation model by sex.

	Total effect		Indirect effect		Mediation ratio (%)
	β	95%CI	β	95%CI	
**Men**	**−0.173**	**−0.237 to −0.112**	**0.038**	**0.010 to 0.068**	**22.0**
Dietary risk			−0.006	−0.018 to 0.003	3.5
Alcohol consumption			0.000	−0.005 to 0.001	0.0
Smoking			0.004	0.000 to 0.014	2.3
Sedentary behavior			**0.041**	**0.025 to 0.062**	**23.7**
Equalized income			0.001	−0.018 to 0.018	0.6
Mental health			−0.002	−0.013 to 0.002	1.2
**Women**	**−0.144**	**−0.198 to −0.019**	**−0.044**	**−0.070 to −0.019**	**30.6**
Dietary risk			**−0.023**	**−0.037 to −0.013**	**16.0**
Alcohol consumption			**−0.006**	**−0.014 to −0.001**	**4.2**
Smoking			0.003	0.000 to 0.010	2.1
Sedentary behavior			**0.012**	**0.004 to 0.023**	**8.3**
Equalized income			**−0.022**	**−0.041 to −0.004**	**15.3**
Mental health			**−0.007**	−**0.019 to −0.001**	**4.9**

*Results adjusted for age. Estimates statistically significant at *p* < 0.05 in bold.

In men, a higher level of education significantly predicted decreased dietary risk (β = −0.14; *p* < 0.001), while it predicted increased smoking behaviors (β = 0.08; *p* < 0.05), sedentary behavior (β = 0.24; *p* < 0.001), and income (β = 0.30; *p* < 0.001; Fig. 1). In women, a higher level of education significantly predicted decreased dietary risk (β = −0.16; *p* < 0.001), and smoking (β = −0.17; *p* < 0.001), while it was associated with higher alcohol intake (β = 0.10; *p* < 0.05), sedentary behavior (β = 0.08; *p* < 0.05), income (β = 0.34; *p* < 0.05), and mental health (β = 0.10; *p* < 0.05; Fig. 2).

The direct effect of education on adiposity was found in both sexes, β = −0.21, 95% CI [−0.277, −0.149] in men, and β = −0.10, 95% CI [0.158, −0.044] in women. The total indirect effect was also statistically significant for both sexes but showed varying directions: β = 0.038, 95% CI [0.010, 0.068] in men, and β = −0.044, 95% CI [−0.070, −0.019] in women (Table 4).

In men, the effect of education on adiposity was significantly mediated by sedentary behavior (β = 0.041; 95% CI [0.025–0.062]), with a mediation ratio of 23.7%. Other mediators did not show statistically significant indirect effects (Table 4). In women, the effect was mediated by dietary risk (β = −0.023, 95% CI [−0.037, −0.013]), alcohol intake (β = −0.006; 95% CI [−0.014, −0.001]), sedentary behavior (β = 0.012, 95% CI [0.004,0.023]), income (β = −0.022; 95% CI [−0.041, −0.004]), and mental health (β = −0.007; 95% CI [−0.019, −0.001]). The total mediation ratio was 30.5%.

## Discussion

The purpose of this study was to investigate mediators of educational disparities in adiposity in the Czech middle-aged population. The mediating pathways were assessed separately for men and women, which allowed for evaluating possible sex differences. Men presented worse quality of lifestyle but reported higher income and lower burden of mental health difficulties, as compared to women. In both sexes, education was associated with almost all potential mediators except for alcohol consumption and mental health in men; however, not all these mediators were significantly associated with adiposity; thus, the observed indirect effects considerably differed between sexes.

In women, increased adiposity in those with lower educational levels seems to be the consequence of a mixture of inappropriate diet and suffering from economic disadvantage. Among the identified mediators, dietary risk and income had the largest mediation ratio of 16% and 15%, respectively. On the contrary, in men, none of the assessed mediators could partially explain the reversed gradient between education and adiposity. Only pathway via sedentary behavior had a significant indirect effect but showed the association in the opposite direction than for the direct effect. In other words, sedentary behavior in men did not partially explain why men with lower education presented increased adiposity despite the considerable strength of the mediation ratio (24%). The opposite mediation effect of sedentary behavior was also observed in women, but with a lower magnitude (8%).

The opposite indirect effect found in sedentary behaviors suggests that in both sexes, the protective potential of higher education in adiposity risk is decreased by the influence of a sedentary lifestyle, which is more prevalent in higher educational groups. Based on the previous studies, increased sedentary behaviors are generally more prevalent in men [25] than women [25] and in higher socioeconomic positions, which may be explained by higher demands for sitting-based tasks in higher-status occupations [26]. Sedentary behavior is an important risk factor in modern society. An increasing trend in sedentary behaviors observed in the last 20 years in European countries [25] could extend the burden of increased adiposity into higher socioeconomic strata and thereby suppress the protective potential of higher education. Lifestyle interventions should therefore emphasize strategies for reducing sitting time in the population, with a special emphasis on higher socioeconomic groups.

It is noteworthy that mediators investigated in the current study explained only one-third of the total association between education and adiposity in women and one-fifth in men. It seems that there are other factors not included in our analysis whose mediating potential exceeds the investigated variables. Lower education may be reflected in reduced knowledge about health and limited health literacy [27], which is the individual ability to obtain, understand, evaluate, and apply health information [28]. One previous study, including 88,384 participants of Lifelines Cohort Study [29] assessed the mediation potential of health literacy in the educational disparities in metabolic syndrome. The study found that health literacy mediated 7.1% of the association in men and 5.9% in women. Besides the mediating role of health literacy, the study also found that self-management, defined as the individual ability to realize and sustain well-being, contributed to educational differences in metabolic syndrome in both sexes [29].

Moreover, although our study investigated a wide spectrum of health behavior factors, we did not consider all individual aspects. For example, our study investigated dietary risk as the frequency of consumption of specific food groups, which as a mediator explained 16% of the total effect in women but did not emerge as a significant mediator in men. This does not mean that an individual diet does not contribute to obesity development, but individual diet results from a variety of factors, including daily eating behavior, cooking habits, or portion size. One case-control study from France [30] including 318 obese and 371 non-obese participants, assessed the mediation potential of eating attitudes, circumstances, and behaviors in the association between socioeconomic status and obesity. Among 10 factors tested, the results showed a significant mediation effect of eating off a large plate, eating at night, and uncontrolled eating [30].

Educational disparities are not reflected only in individual habits and abilities but are also closely related to the disadvantaged life environment. The availability of healthy options is often higher in affluent neighborhoods compared to socially disadvantaged areas [31]. Besides that, the external environment influences adiposity both at the level of the social environment as well as the physical environment. The social environment can work as an obesogenic environment that pushes persons to make choices and decisions culturally perceived as normal and accepted even though they might not be healthy [32]. On the contrary, the physical environment does not modify individual habits but can directly affect adiposity risk as a biological response to exposure to chronic stress [33], endocrine-disrupting chemicals [34], or air pollution [35].

The findings of our study suggest that future research should focus on an even broader investigation of external as well as internal factors, which may have the potential to complement the mediators investigated in the current study to better explain why increased adiposity is more prevalent in groups with lower education. The identification of various pathways will help focus prevention activities on specific behaviors or characteristics and improve cardiometabolic health in the disadvantaged groups of the population.

The major strength of the present study is the complex approach to adiposity assessment, including several anthropometric and bioimpedance measures, and the investigation of a wide spectrum of potential individual mediators. Moreover, by analyzing and reporting results separately for men and women, the study acknowledges and accounts for potential gender variations in the observed relationships. However, there are some limitations of this study that should be mentioned. First, the study’s cross-sectional design does not allow causality evaluation; thus, the direction of the associations set in the multiple mediation model was constructed based on previous research evidence, and a reverse causation bias might occur. Second, the study sample only included a city-based population; thus, the study findings should not be generalized beyond the urban population. Third, investigated mediators were mostly self-reported; therefore, reporting bias, including underestimation, might occur. However, objectively measured data on these factors were not available, and questionnaire based for behaviors such as smoking, or alcohol consumption are widely accepted. Additionally, the version of FFQ used in the current study has not been previously validated in different study; however, the content has been developed in accordance with previous literature recommendations for FFQ development and utilization [16, 36].

Based on our findings, we conclude that educational disparities in adiposity and related mediators might differ by sex. The increased adiposity in women with lower educational levels is likely driven by dietary risky behaviors and lower income, but we did not observe a mediator which contributes to the inverse gradient between education and adiposity in men. However, our study identified sedentary behaviors as a risk factor possibly reducing the protective potential of higher educational levels in both sexes, with a higher magnitude in men. This finding suggests that reducing sedentary time should be sufficiently targeted in public health strategies to reduce the burden of increased adiposity in the population. Future studies should investigate a broader spectrum of potential mediators, including knowledge-related individual factors as well as components of the external social and physical environment.

### Supplementary information

Supplementary figure 1

Supplementary table 1

## Data Availability

The data that support the findings of this study are available from ICRC - FNUSA but restrictions apply to the availability of these data, which were used under license for the current study, and so are not publicly available. Data are available upon reasonable request and with permission of ICRC-FNUSA.
