# Supplementary material for: The explanation of educational disparities in adiposity by lifestyle, socioeconomic and mental health mediators: a multiple mediation model
Source: Eur J Clin Nutr. 2024 Jan 20;78(5):376–83. doi: 10.1038/s41430-024-01403-1 (PMC11078717; doi:10.1038/s41430-024-01403-1)

Supplementary figure 1: The latent variable representing adiposity, separately for men (A) and women (B). Model fit in men: $\chi^2(1) = 21.2$, $p < 0.001$, CFI = 0.996, RMSEA = 0.144, 90% CI RMSEA (0.095, 0.200). Model fit in women: $\chi^2(1) = 34.7$, $p < 0.001$, CFI = 0.995, RMSEA = 0.169, 90% CI RMSEA (0.124, 0.219). All reported standardized coefficients significant at p<0.001.

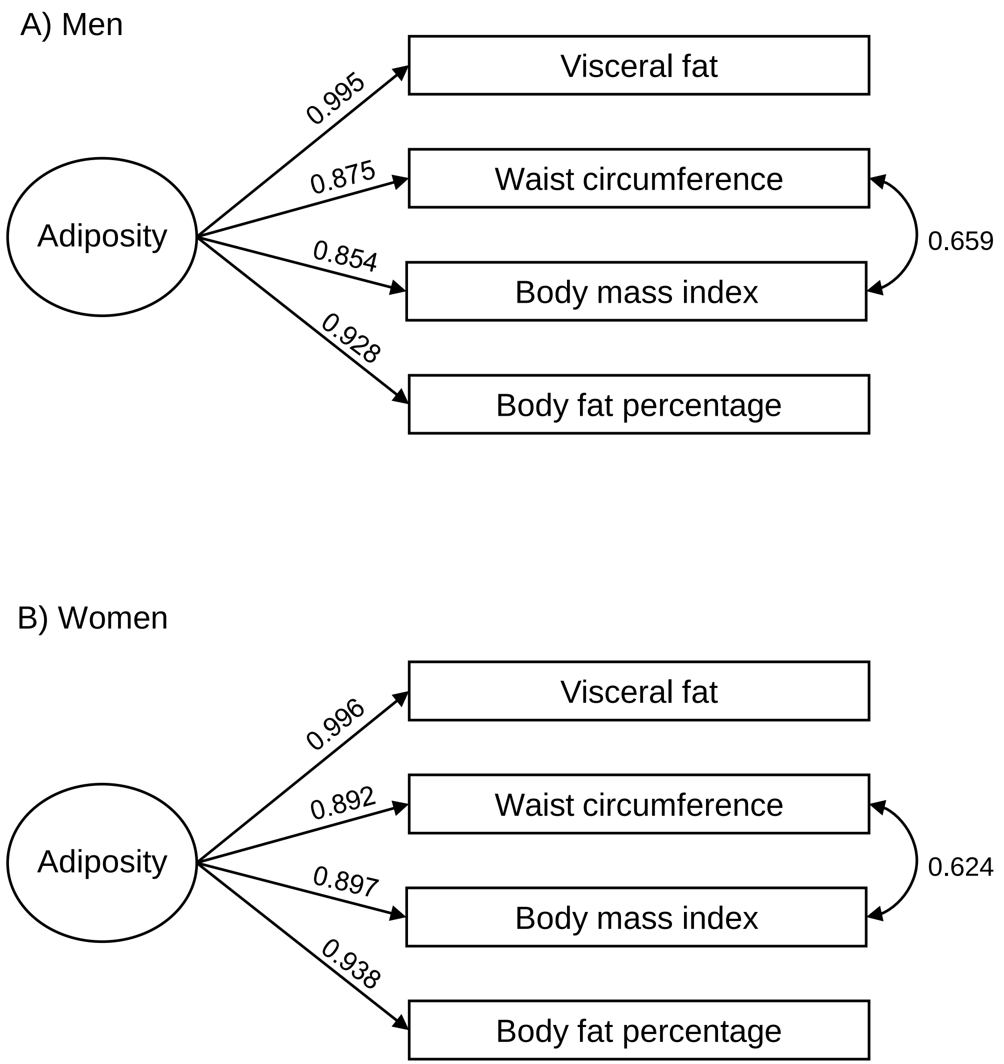

Supplement: Supplementary file 1 — Supplementary figure 1 [file 41430_2024_1403_MOESM1_ESM.pdf]
