# Supplementary material for: The explanation of educational disparities in adiposity by lifestyle, socioeconomic and mental health mediators: a multiple mediation model
Source: Eur J Clin Nutr. 2024 Jan 20;78(5):376–83. doi: 10.1038/s41430-024-01403-1 (PMC11078717; doi:10.1038/s41430-024-01403-1)
Supplement: Supplementary file 2 — Supplementary table 1 [file 41430_2024_1403_MOESM2_ESM.pdf]

Supplementary table 1: Correlation between potential mediators

	Dietary risk behaviors	Alcohol intake	Smoking	Sedentary behavior	Equalized income	Stress	Depression	Quality of life
**Dietary risk behaviors**	MEN	.01	**-.11****	**.09****	**-.08****	**.10****	**.06****	**-.08****
**Alcohol intake**	**.10****		-.07**	**.10****	**.10****	-.00	-.01	.05
**Smoking**	-.07**	-.06		-.06**	.03	-.00	-.05	**.06****
**Sedentary behavior**	-.01	-.04	-.01		**.18*****	.04	.05	**-.07****
**Equalized income**	**-.08****	.03	.02	**.14*****		-.06	**-.13*****	**.18*****
**Stress**	.04	.06	-.28	**-.07****	**-.10****		**.56*****	**-.35*****
**Depression**	.00	.01	-.03	**.10****	**-.08****	**.56*****		**-.42*****
**Quality of life**	**-.07****	.02	-.07	-.04	**.18*****	**-.32*****	**-.42*****	WOMEN

Correlation coefficients below diagonal for men, above diagonal for women. **p<0.05; ***p<0.001
